# Identifying Spatial Matching between the Supply and Demand of Medical Resource and Accessing Carrying Capacity: A Case Study of Shenzhen, China

**DOI:** 10.3390/ijerph19042354

**Published:** 2022-02-18

**Authors:** Jiansheng Wu, Jiayi Fu, Hongliang Wang, Yuhao Zhao, Tengyun Yi

**Affiliations:** 1 Peking University (Shenzhen) Laboratory for Urban Future, School of Urban Planning and Design, Peking University Shenzhen Graduate School, Shenzhen 518055, China; wujs@pkusz.edu.cn; 2 Key Laboratory for Earth Surface Processes, Ministry of Education, College of Urban and Environmental Sciences, Peking University, Beijing 100871, China; zhaoyh2017@pku.edu.cn; 3 Key Laboratory for Urban Habitat Environmental Science and Technology, School of Urban Planning and Design, Peking University, Shenzhen 518055, China; fujiayi@stu.pku.edu.cn (J.F.); tengyun_yi@163.com (T.Y.); 4 School of Public Administration, Inner Mongolia University, Hohhot 010070, China

**Keywords:** medical resources, supply–demand balance, coupling coordination degree model, two-step floating catchment area method (2SFCA)

## Abstract

Previous Studies, such as the evaluation of the supply of and demand for regional medical resources and carrying capacity assessments, require further development. This paper aims to evaluate the carrying capacity and spatial distribution of medical resources in Shenzhen from the perspective of supply and demand, and to conduct a time-series variation of the coupling coordination degree from 1986 to 2019. The two-step floating catchment area method was employed to quantify the carrying capacity and coupling coordination degree method and spatial autocorrelation analysis were applied to analyze spatial distribution between supply and demand. The results were as follows. (1) The carrying capacity index in more than 50% of the districts was classified as low-grade. The percentage of regions with good grades was 8.27%. The regions with a high carrying capacity were distributed in the central and southeastern areas. (2) The coupling coordination continued to rise, increasing from 0.03397 in 1986 to 0.33627 in 2019. (3) The level of supply and demand for medical resources in Shenzhen increased from 1986 to 2019, and the highest degree of compatibility between the supply and the population size was largely concentrated in the western and eastern regions. This research can provide a theoretical reference for Shenzhen to rationally plan medical resources and improve the carrying capacity of medical resources.

## 1. Introduction

Against the backdrop of rising urbanization and an increasing urban population, the demand for public health services has increased dramatically. Medical resources are critical to ensuring the provision of sound public medical services in cities [1,2,3]. The outbreak of the new coronavirus in 2019 has aroused widespread concern among populations and academic circles. In order to respond to such sudden public health crises, improving the public health system and rationally allocating medical resources have become the focus of the governments of various countries [4,5]. However, in mega-cities, the mis-match between the supply of and demand for medical resources is significant due to the more obvious increase in population, and presents a social issue that governments need to urgently address to safeguard people’s livelihoods and stabilize society [6,7,8]. Research related to medical resources tends to focus on issues related to the acces-sibility and equity of medical resources. For example, the accessibility of medical re-sources is used as an indicator to evaluate the spatial distribution balance and equality of medical resource allocation in a particular region, which can thereby identify problems related to the uneven distribution and insufficient supply of medical resources in certain areas [9,10,11]. Some of these studies used improved accessibility calculation methods, such as the two-step floating catchment area method, gravity models, and geographically weighted regression models [12,13,14,15]. Other studies have conducted accessibility studies for specific contexts or regions, and explored changes in the equity and accessibility of medical resources under the impact of hospital referral reforms [16]. Moreover, appropriate accessibility evaluation models have been utilized for specific regions, such as those with a low population density and desert areas [17]. On the supply side, some studies have also explored the supply and allocation of medical resources within the context of the 2019 novel coronavirus (COVID-19) pandemic, in an effort to respond to the associated public health crisis [18,19]. The above studies on public services have promoted the development of the field from the perspectives of equity, accessibility, and the relationship between different types of public service. Furthermore, they provide a methodological reference for studies related to medical resources and medical services in terms of model quantification, spatial analysis, coupling, and coordination. However, research has neglected the relationship between the supply of and demand for medical resources in space and quantity, and the spatial distribution of medical resource carrying capacity needs to be further explored.

Shenzhen is a cosmopolitan city on the southeast coast of China, and its population density is much higher than that of similar first-tier cities, such as Beijing, Guangzhou, and Shanghai [20]. Given Shenzhen’s rising economic level, growing number of permanent residents, and changing floating population, as well as its increasing elderly population, residents’ demand for medical resources is showing a rising and diversified trend. At present, Shenzhen is faced with the problem that the per capita index of medical resources is not as optimal as that of similar first-tier cities; the total amount of medical resources in the city, especially high-quality resources, is relatively insufficient, and the allocation and utilization of medical resources is unbalanced [21]. Based on the above background, this paper quantified Shenzhen’s carrying capacity of medical resources and analyzed the spatial distribution of supply and demand, which is helpful to the provision of a theoretical framework for rational planning in the area of medical resources for local areas, and has a certain reference value for other cities and regions. Therefore, this study evaluated the carrying capacity and spatial distribution of medical resources in Shenzhen from the perspective of supply and demand through the two-step floating-catchment-area method, conducted a time-series variation of the coupling coordination degree, and analyzed the spatial distribution of supply and demand by applying the coupling coordination degree method and spatial autocorrelation analysis. More precisely, the following research questions were addressed. (1) We explored the level and spatial distribution characteristics of Shenzhen’s carrying capacity for medical resources. (2) We quantified the relationship between the supply of and demand for Shenzhen’s medical resources. (3) We analyzed how supply and demand match up spatially.

## 2. Data and Methodology

### 2.1. Study Area

**Figure 1:**
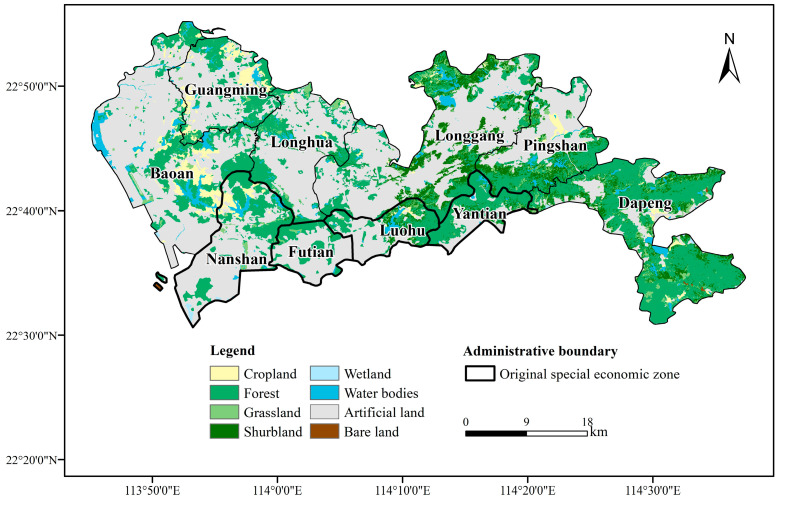
The administrative division of Shenzhen.

**Figure A1:**
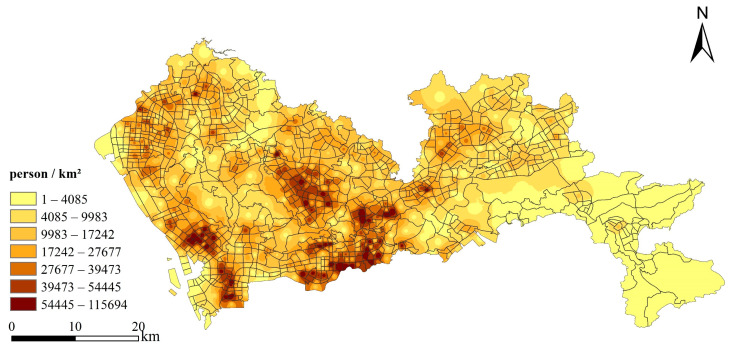
Population density of Shenzhen in 2019.

Shenzhen city (113°46′–114°37′ E, 22°27′–22° 52′ N), located in the southeastern coastal region of China, is one of the constituent cities of the Guangdong–Hong Kong–Macao Greater Bay Area, a world-class city cluster with a developed economy. The total area of the city is 1997.47 km^2^. In 2019, the total population of Shenzhen was 18,496,000 (Figure A1). The Shenzhen Special Economic Zone (SEZ) was established in 1980 and consisted of four administrative districts (Luohu, Futian, Nanshan and Yantian). The original SEZ territory was extended to include the whole city in 2010 (Figure 1). At present, Shenzhen is faced with a relative shortage of medical resources, especially quality resources, in addition to pressure on medical services to meet the rapidly growing demand of residents for medical services [22]. In this article, the study area is based on the traffic analysis zones (TAZ), excluding enclaves and islands.

### 2.2. Data Sources

(1) Sixty-seven public hospitals in Shenzhen and relevant information were obtained from the Shenzhen Municipal Government (http://www.sz.gov.cn/hospital/ssyy/rmyy/jggk/index.html, accessed on 1 May 2021) and the Shenzhen Municipal Health Commission (http://wjw.sz.gov.cn/bmfw/wycx/fwyl/yycx/index.html, accessed on 1 May 2021) websites. (2) Data related to medical resources included the hospital grade, number of beds, number of personnel, total medical treatments, number of outpatients, emergency visits, and the number of inpatients and discharged patients. (3) Socio-economic and health care data related to the period 1986–2019 were collected from the Shenzhen Statistical Yearbook (http://www.sz.gov.cn/cn/xxgk/zfxxgj/tjsj/tjnj, accessed on 1 May 2021). (4) Population data were processed from mobile phone signaling data that were divided according to the system. Mobile phone signaling data were obtained from the Shenzhen Planning, Land and Property Information Center project in October and November 2019. Road network data were obtained from Open Street Map by applying road categories that included, for example, urban trunk roads, urban secondary roads, and urban feeder roads. These data were then used to carry out the origin–destination (OD) cost matrix analysis and network analysis. Hospital point data were sourced from 2018 POI data from the Gaode Map Open Platform.

### 2.3. Research Methodology

#### 2.3.1. Research Framework

**Figure 2:**
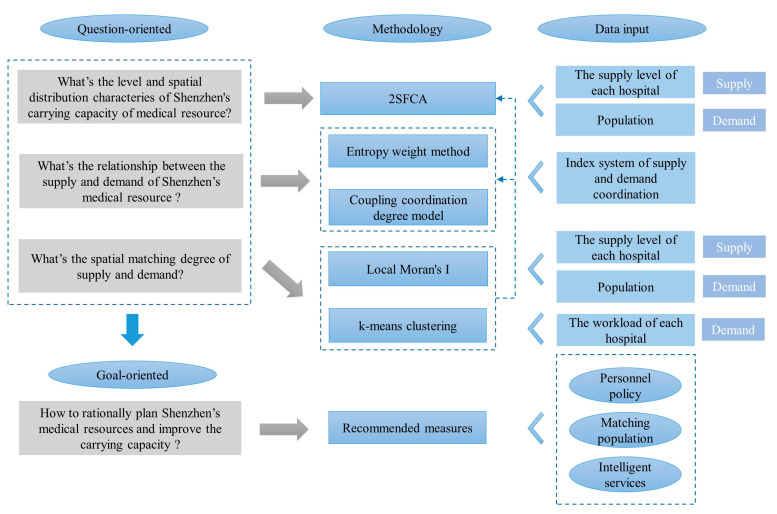
Research framework.

This study adopted a four-step approach (Figure 2), as follows: (1) we established a supply-and-demand coordination evaluation index system; (2) we quantified and spatially visualized the carrying capacity of medical resources; (3) we calculated the coupling coordination degree; and (4) we performed a matching analysis of the spatial supply and demand of medical resources. This paper utilized a spatial autocorrelation tool (local Moran index) to identify high- and low-value spatial clusters of supply levels and populations in Shenzhen [23,24]. To further refine the spatial distribution of medical resources in terms of demand, this study collected workload indicators for 64 hospitals that largely corresponded to hospital supply points, including total visits, outpatient visits, emergency visits, inpatient visits, and hospital discharges. The K-means clustering method was employed [25,26], and the number of clusters was four, based the elbow method. The elbow method was widely used to determine the optional numbers of the clusters, since it is simple to implement and verify through visualization [27,28]. All four clusters were classified according to the average workload of the hospitals. The clusters were divided into four groups, which were ranked from high to low, corresponding to the requirements of level 1 to level 4.

#### 2.3.2. Establishment of an Assessment Indicator System and Determination of Index Weight

This study established a comprehensive index system to evaluate the coordination of the supply of and demand for medical resources in Shenzhen (Table 1), which provided the basis for determining the coupling coordination degree of supply and demand. Indicators were selected based on existing studies and available data, and on the principle that they quantified supply and demand and were complete in the data set. According to previous relevant studies, the indicators could reflect the supply and demand to a large extent [29,30].

In this research, all of the indicators were positive. Based on this, the weighting method applied in the study was the entropy method. As an objective weighting method, it determines objective weights based on the magnitude of the variability of indicators, which avoids bias caused by human factors [31]. To eliminate the effects of dimensional and scale differences of the original data, the data were standardized and calculated as follows:

(1) $$r_{ij=}\frac{V_{i}-V_{min}}{V_{max}-V_{min}}$$

where $r_{ij}$ represents the standardized data for indicator *j* in year *i,* $V_{i}$ represents the original data, while $V_{imax}\;$and$\;V_{imin}$ indicate the maximum and minimum value of the indicator, respectively.

The entropy value method was used to determine the index weight:

(2) $$w_{i}=\frac{1-H_{i}}{m-\sum_{i=1}^{m}H_{i}}$$

where $H_{i}=$ $-\frac{1}{\ln n}\overset{n}{\underset{j=1}{\sum }}f_{ij}\ln f_{ij}$, represents the entropy of indicator *i^th^* index $H_{i}$; $f_{ij}=\frac{r_{ij}}{\sum_{j=1}^{n}r_{ij}}$ represents the weighting of *j^th^* indicator in the year *i^th^* $f_{ij}$; and $w_{i}$ expresses the calculated index weight, such that $0\leq w_{i}\leq 1$.

#### 2.3.3. Two-Step Floating Catchment Area Method (2SFCA)

The study adopted the two-step floating catchment area method to measure the carrying capacity of medical resources in Shenzhen and visualize it spatially. Although the 2SFCA has been widely used to evaluate the accessibility of public service facilities due to its operability and practicality [16,32,33], it also can be applied to assess the matching relationship between supply and demand [34]. The basic idea is to calculate the ratio of supply to demand within the search threshold in two steps. This study applied the “medical resources carrying capacity index” as a comprehensive index to measure the carrying capacity of medical resources, borrowing the characteristics of the three dimensions of the 2SFCA, namely supply, demand, and distance. According to the basic principles of the 2SFCA, and taking into account the distance of the road network, for each demand point, the supply points within its search radius are searched and the supply-demand ratio is weighted and summed up, and then for each demand point, the supply points within its search radius are searched and the supply-demand ratio is weighted and summed up to obtain the carrying capacity of that demand point. The higher the ratio, the higher the carrying capacity of the region.

Furthermore, we considered the level of supply based on each hospital’s current resource status and grade, and constructed a comprehensive evaluation system for the public hospitals in Shenzhen by using three indices, namely, the number of beds, the number of personnel, and the hospital grade. For demand, we used population data in from the traffic analysis zones processed by mobile signaling data as a demand indicator [35]. The population data was from the total population, including the resident population, 67%, and the mobile population, 33%. After data cleaning, sample expansion and other pre-processing, the resident population, working population, and mobile population were identified according to the population classification analysis model. TAZ is a management unit for the study of citizen travel generation and travel division by the city government in transportation planning, its internal homogeneity is required in the process of division, natural barriers such as railroads and rivers should be used as zoning boundaries in the boundary division as much as possible, and administrative boundaries should be retained as much as possible boundaries [14,36,37]. Therefore, the land use, economic, and social characteristics within the traffic cell are relatively consistent, and there is a high consistency of traffic conditions within the same zone, as has been demonstrated in articles using Shenzhen as the study area [38]. In the context of today’s big data, the use of mobile signaling data to identify the population density of a cell can be timelier and more accurate, thus overcoming the shortcomings of traditional street-based statistics [39].

Prior to the use of 2SFCA, this study obtained the nearest distance from the demand point to the supply point using the OD cost matrix solver in the ArcGIS network analysis tool (Environmental Systems Research Institute, RedLands, The United States of America). The maximum nearest distance between each demand point and each supply point was used as the threshold.

In the first step, each supply point *j* (hospital point) was searched for demand points *k* (TAZ surface centroids) within its search radius and the supply-to-demand ratio; that is, we calculated the ratio of the supply level to the number of people. In the second step, for each demand point *i*, the supply points were searched within a threshold (*d*_0_) from location *j* and the supply-to-demand ratio was summed to obtain the medical resource carrying capacity of each demand point. The spatial differentiation of the carrying capacity and the spatial pattern can be obtained through this a method. The equation used in the first step is as follows:

(3) $$R_{j}=\frac{s_{j}}{\Sigma_{k\in \left\{d_{kj}\leq d_{0}\right\}}D_{k}}\;$$

where $R_{j}$ represents the medical services supply to the population ratio of hospital location (supply point) *j*, whose search threshold falls within the catchment ($d_{kj}\leq d_{0}$); $D_{k}$ is the scale of demand point *k*, whose search threshold falls within the catchment ($d_{kj}\leq d_{0}$); $S_{j}$ is the scale of supply at location *j*; and $d_{kj}$ is the distance between *k* and *j*.$\;d_{0}$ denotes the search threshold calculated in the OD cost matrix. The formula used in the second step is as follows:

(4) $$A_{i}^{F}=\underset{j\in \left(d_{ij}\leq d_{0}\right)}{\sum }R_{j}=\underset{j\in \left(d_{ij}\leq d_{0}\right)}{\sum }\left[\frac{s_{j}}{\Sigma_{k\in \left(d_{k}\leq d_{0}\right)}D_{k}}\right]\;$$

where $A_{i}^{F}$ represents the medical resources carrying capacity at the TAZ. A larger value of $A_{i}^{F}$ indicates a higher carrying capacity of medical resources at a location. $d_{ij}$ is the distance between *i* and *j*.

Given that residents’ willingness to travel and the probability of hospital attendance decrease as the distance increases, this study selected the Kernel density function to model distance decay. The kernel density distance decay function is concave function. When the distance between the supply point and the demand point is small, the accessibility decays slowly as the distance increases; when the opposite is the case, it increases [40,41]. The calculation formulas are as follows:

(5) $$R_{j}=\frac{s_{j}}{\Sigma_{k\in \left\{d_{kj}\leq d_{0}\right\}}G\left(d_{kj}\right)D_{k}}\;$$

(6) $$G\left(d_{kj}\right)=\frac{3}{4}\left[1-{\left(\frac{d_{kj}}{d_{0}}\right)}^{2}\right],\;d_{kj}\leq d_{0}\;$$

where $G\left(d_{kj}\right)$ is Kernel density function. The decay rate of distance continues to increase with the increase of distance $d_{kj}$ between two points.

#### 2.3.4. Coupling Coordination Degree Method

To effectively reflect the coordination level and the synergistic effect between supply and demand, this paper used the coupling coordination degree method to evaluate the complex relationship between the two systems [42,43]. *X_i_* and *Y_i_* measured the supply and demand level, and the indicators are shown in Table 2.

To define the degree of coordination, this study adopted the concept of coupling coordination levels based on existing studies [45,46,47]. The carrying capacity index of each TAZ was graded in ArcGIS according to the natural breakpoint method, as can be seen from Table 3 [48]. The natural breakpoint classification method achieves natural clustering by minimizing the intra-group spacing and maximizing the inter-group spacing according to the statistical nature of the data [49].

## 3. Results

This section is divided by subheadings. It should provide a concise and precise description of the experimental results, their interpretation, and the experimental conclusions that can be drawn.

### 3.1. Analysis of the Carrying Capacity of Medical Resources in Shenzhen

According to the results of the 2SFCA, the carrying capacity index of each TAZ was classified into five grades of carrying capacity, ranked from higher to lower: higher, high, middle, low, and lower (Table 4). It can be seen that more than 90% of the carrying capacity index of the TAZs was between 0.055 and 0.455, which meant that they fell within the range of low-to-lower bearing capacity. The percentage of TAZs with low and lower carrying capacity was 51.71%, which was comparable to the percentages associated with carrying capacity levels that were middle or above.

**Figure 3:**
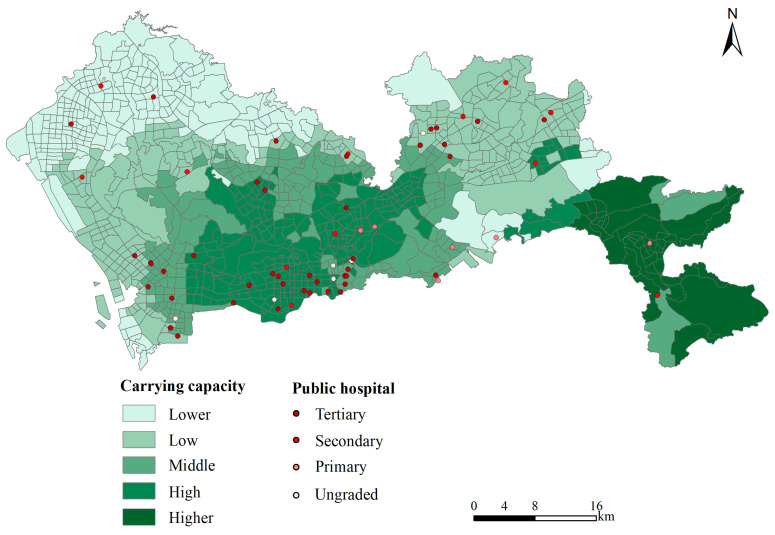
Spatial distribution of Shenzhen’s carrying capacity of medical resource.

Spatially, the carrying capacity of Shenzhen’s medical resources was distributed in a stepwise pattern from northwest to southeast (Figure 3). The higher carrying capacity areas were mainly located in the central and eastern parts of Shenzhen, primarily within the former SEZ (Figure 1). The lower carrying areas were located in the north-western part of the city. By administrative district, Futian District and the eastern part of Nanshan District had the best carrying capacity. The areas with lower carrying capacities were mainly located in Baoan District, Guangming New Area, and the northern part of Longhua New Area. Overall, the carrying capacity of medical resources in Shenzhen was unevenly distributed, both spatially and in terms of quantity. The areas with the highest carrying capacity for medical resources were distributed in the central and south-eastern areas, while the north-western, northern, and southern edges had a lower carrying capacity.

**Figure 4:**
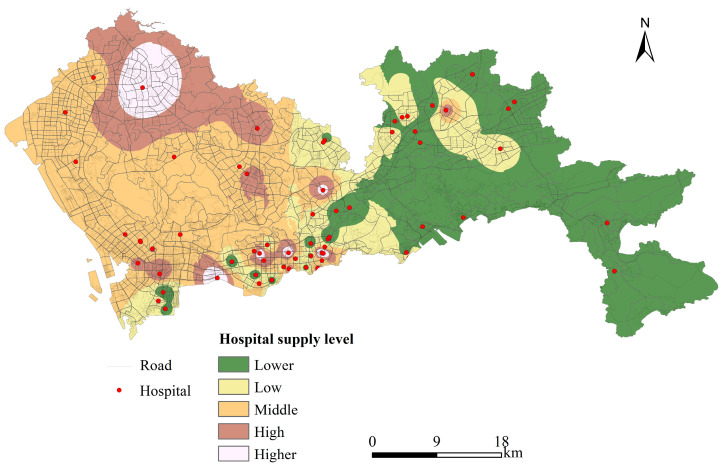
Spatial distribution of road network and hospital supply level in Shenzhen.

The spatial distribution of the supply level (comprehensive scoring of the hospital’s current resource status and grade based on the entropy weighting method) of public hospitals in Shenzhen was obtained by spatially interpolating the supply scores of each public hospital point using inverse distance weighted (IDW) interpolation (Figure 4). The spatial distribution of public hospital supply levels in Shenzhen varied significantly from the west to the east, and showed a high-to-low distribution. Hospitals with higher supply levels were concentrated in the southwestern part of Shenzhen, namely, the southern part of Nanshan District, Futian District, and Luohu District. Although Longgang District also had more tertiary hospitals, the level of supply was not as high as that of Futian and Luohu Districts.

### 3.2. Analysis of the Coordinated Development of Supply and Demand

**Figure 5:**
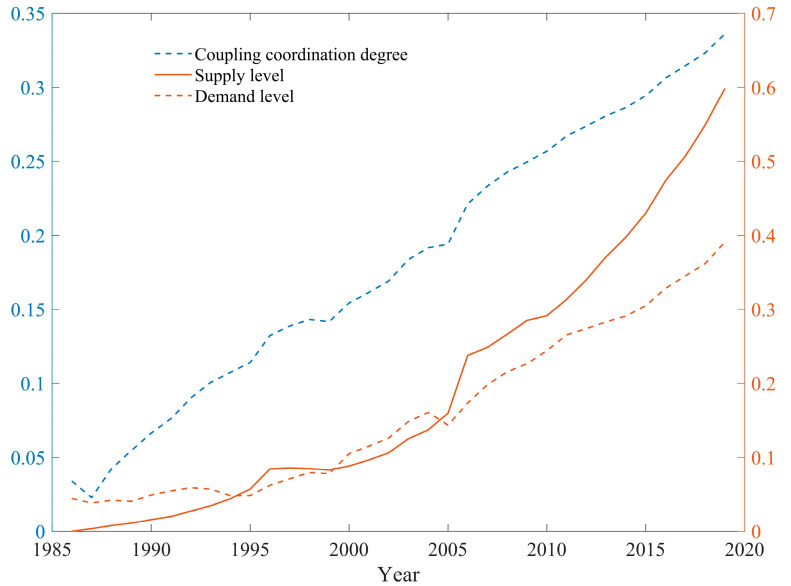
The change in the coupling coordination degree of the supply of and demand for medical resources in Shenzhen from 1986 to 2019.

The supply and demand coordination evaluation index system (Table 1) was applied in the coupling coordination degree model. As can be seen from Figure 5, from 1986, the coupling coordination degree between supply and demand for medical resources in Shenzhen continued to rise, increasing from 0.03397 in 1986 to 0.33627 in 2019 (Table A1). In 2019, the coordination degree was on the verge of dislocation, and the overall trend shows a continued progression towards coordination. More recently, the supply level and demand level have continued to rise: since 2005, the supply level has been slightly higher than the demand level, which indicates that supply has exceeded demand, and the gap between the two has shown an increasing widening trend.

**Figure 6:**
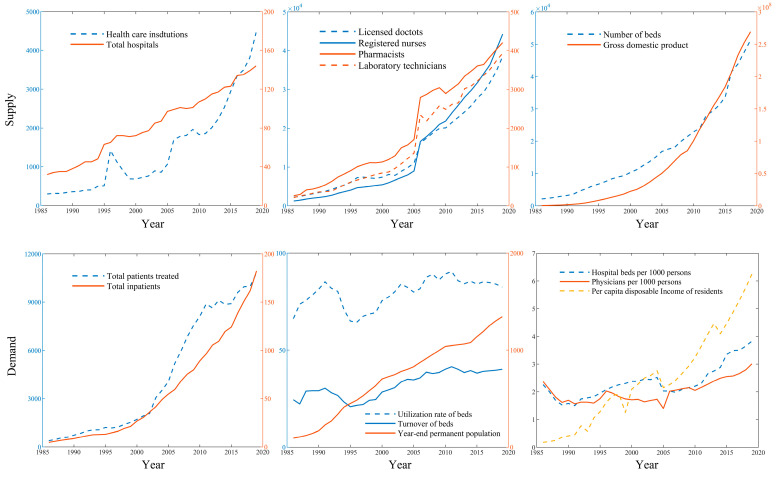
Changes in the supply of and demand for medical resources in Shenzhen from 1986 to 2019.

Changes in Shenzhen’s medical resource indicators are shown in Figure 6 (Table A2 and Table A3). On the supply side, from 1986 to 2019, the construction of medical facilities in Shenzhen developed rapidly. The number of health institutions, the number of beds, and the number of licensed physicians increased by 1401.35%, 350.00%, and 1637.44% respectively, while the total number of medical service facilities also increased. With the increase in the level of supply, the structure of medical and nursing staff was optimized and growth was also observed in the supply capacity of hospitals. On the demand side, from 1986 to 2019, Shenzhen’s resident population increased rapidly and people’s living standards improved. In comparison with 1986, the year 2019 saw an increase of 2679.43% and 3793.16% in the total number of medical consultations and hospital admissions, respectively, as well as an increase of 3340.95% in per capita disposable income. At the same time, the number of beds per 1000 population and doctors per 1000 population increased from 2.26 and 2.37 in 1986 to 3.83 and 3.01 in 2019, respectively. The Plan for the Establishment of Medical Institutions in Shenzhen (2016–2020) outlined a plan to increase the supply of medical resources to achieve 4.3 beds per 1000 residents and 2.8 practicing (assistant) doctors per 1000 residents by 2020; in the case of the latter, this target was reached in 2019.

### 3.3. Spatial Matching of Supply of and Demand for Medical Resources

**Figure 7:**
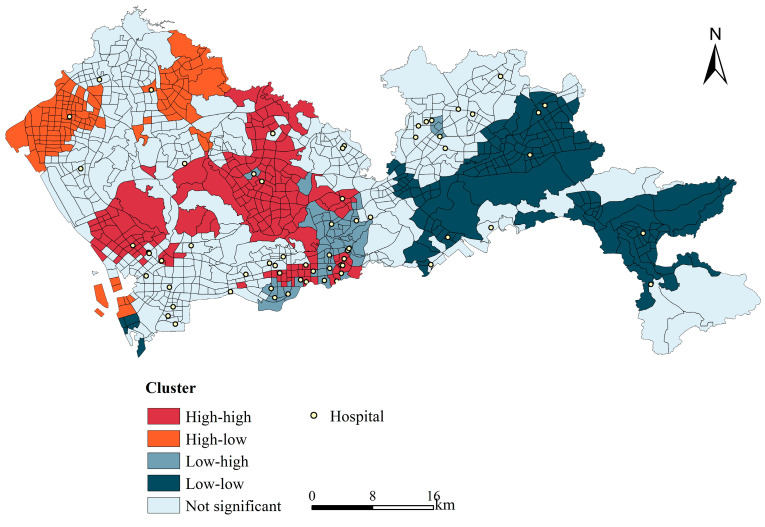
Supply–demand bivariate spatial autocorrelation.

Using the two indicators of supply and population, the bivariate spatial autocorrelation of supply and demand was calculated using Geoda software, and the results passed the significance test at the 0.01 level (Figure 7). The high-high clusters mainly appeared in the west of Shenzhen, concentrated in Longhua District, the southern part of Baoan District, and the northern part of Nanshan District, indicating that hospitals with higher supply levels were located in areas with a higher population distribution. The high-low clusters were mainly concentrated in the north-western part of Shenzhen; that is, in the north-western Baoan District and eastern Guangming New District. In the case of the Baoan District and western Guangming New District, the difference was due to the fact that hospitals with higher supply levels are located in areas that had a lower population. The low-high clusters were mainly located between the high-high and low-low clusters, concentrated in the western part of Longgang District, Futian District, and Luohu District. The low-low clusters were mainly located in the eastern part of Shenzhen, concentrated in Dapeng New District and Pingshan District.

**Figure 8:**
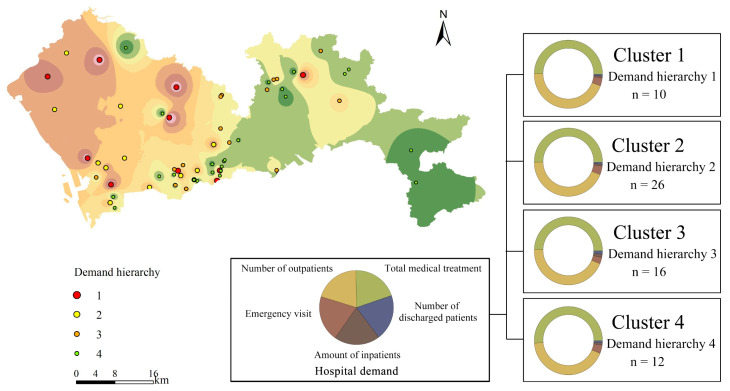
Spatial distribution of the medical resources demand cluster.

**Figure A2:**
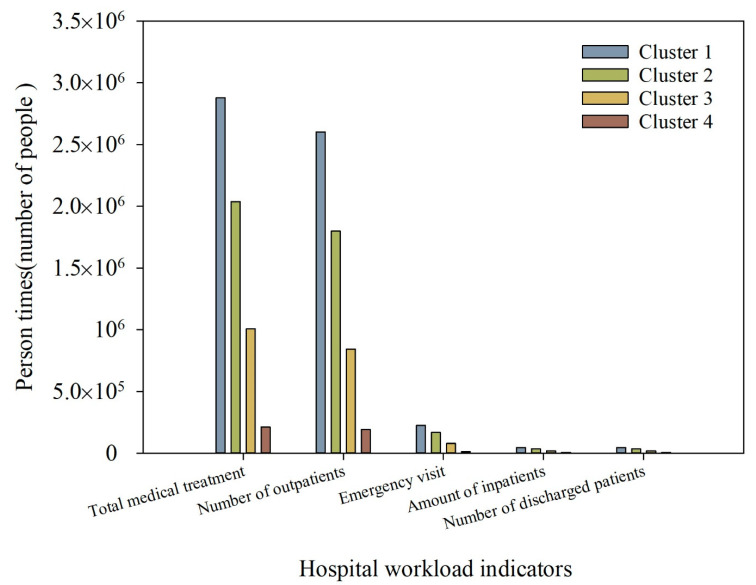
Spatial distribution of medical resource demand cluster.

K-means cluster analysis was performed on the five demand indicators at each hospital site. Through the elbow method, we determined the optimal cluster number as four. The clustering results passed the significance test at the 0.01 level, classifying the hospitals into four groups according to the level of demand, which was ranked from high to low. The mean values of the specific indicators for each group of clusters are shown in Figure A2. The spatial distribution results were obtained by means of spatial interpolation in respect to the total number of consultations indicator (Figure 8). The hospital sites were divided into four classes according to demand by cluster analysis, with class one corresponding to the largest demand. Different colors in the circles correspond to different indicators. It can be concluded that the obvious high-demand area was the central-western part of Shenzhen; the hospitals with high demand were mainly located in Baoan, Longhua, Futian and Luohu districts. The low-demand hospitals were mainly located in the eastern part of Longgang District and Dapeng New District. In terms of the number of clusters, the number of hospitals with demand Levels 1 and 4 was low, accounting for 15.63% and 8.75%, respectively. The proportion of hospitals with Level 2 and Level 3 was 40.63% and 18.75%, respectively.

## 4. Discussion

### 4.1. Historical Developments and Policies Influence Supply and Demand Characteristics

Influenced by the history of regional development and policies, the carrying capacity and level of supply of medical resources in Shenzhen showed wide variations in space. The SEZ was established in 1979, and in 2010 extended to cover the whole city, resulting in an uneven distribution of medical resources within and outside the former SEZ, with medical resources relatively concentrated in the central city. Nanshan, Luohu, Futian, and Yantian District were distributed within the former SEZ, which was developed earlier and had a relatively concentrated distribution of hospitals and a high level of carrying capacity of medical resources [50]. However, the eastern part of Shenzhen (Dapeng New District) presented the highest carrying capacity due to its low population density and corresponding low demand. For the spatial matching of healthcare resources in space, areas outside the former Shenzhen SEZ, such as the low-low agglomeration areas in supply-demand shown in Figure 6, had a relatively late start to their development and utilization, with relatively few healthcare resources, as well as lower population density and less workload at hospital sites. In addition, low-high agglomeration areas were locally distributed in the northeast of Longgang District, where the corresponding population density is high, and the relative demand for medical resources is relatively insufficient. This is related to the population migration since the Shenzhen Special Economic Zone expanded to the city. This was consistent with the conclusions of previous research [22,50].

On the policy side, the significant increase in the number of medical resources was in part related to the ten-year medical reform that began in 2009, which doubled the total amount of health and medical services in the city [21]. At the same time, Shenzhen invited famous hospitals and medical schools to manage hospitals and medical schools in the city, and assembled high-level teams from domestic and abroad in an effort to promote the integrated development of medical and health care, and, to a certain extent, alleviate the difficulties encountered by residents in accessing medical care. According to the 2018 ranking of China’s Health City Index System, Shenzhen ranked third on the dimension “health services”, which highlighted the remarkable effectiveness of the 10 year health care reform. In terms of the level of demand, the increase in various medical resource demand indicators was closely related to economic development and the improvement of people’s living standards in Shenzhen since the reform and opening up of the 1980s. The significant upward trend in some health care resource indicators after 2005 is related to the change of statistical data port. The Shenzhen Municipal Health Commission publicly stated in its health statistics executive summary that the statistical caliber of the number of personnel before and after 2006 was the number of permanent staff and staff on duty, respectively. According to the Plan for the Establishment of Medical Institutions in Shenzhen (2016–2020), the change in residents’ demand for healthcare was reflected in the multi-level, diversified, and personalized demand for medical services, which were characterized by the need for greater optimization with respect to the allocation of medical resources. In the context of the industrial structure transformation, the population was gradually moving to areas outside the former SEZ. In addition, these imbalances between supply and demand had a tendency to intensify; uneven distribution outside and inside the former SEZ needs to be addressed. While considering the present situation of medical reform, it is critical to improve the allocation of medical resources and increase their utilization rate.

### 4.2. Rationalization of Data and Methods

This study used mobile signaling data as population data, given the current widespread use of smart phones, as well as the wide distribution of signal towers providing communication and Internet services to cell phone users. The signaling data were used to record users’ spatial and temporal information. Mobile signaling data have the characteristics of large volume and high dynamism, which can accurately reflect the dynamic changes in the population of Shenzhen. At the same time, we selected a research method that corresponded to the scientific question. For exploring the spatial distribution of the carrying capacity of medical resources in Shenzhen, the 2SFCA method focused on the supply–demand ratio was chosen. The method did not use a single indicator of the number of beds or the number of personnel as a measure of the supply level, but replaced it with a comprehensive score of three indicators: hospital grade, number of personnel, and number of beds, and added the factor of distance from the supply point and the demand point to the road network, taking into account the distance decay. Previous studies used the same 2SFCA method to evaluate the accessibility of medical resources using Shenzhen city as the study area, incorporating important factors affecting accessibility, such as transportation modes and online maps. These studies concluded that the southeast region has the weakest spatial accessibility [51,52]. This is a different starting point from our study, which used the 2SFCA method to measure the carrying capacity, so the results obtained were more varied.

In addition, in order to explore the dynamic change process of supply and demand levels and coupling coordination degree from time series, we selected specific evaluation indicators to construct the index system and adopted the coupling coordination degree model. To assess and visualize the spatial matching degree between supply and demand, we performed bivariate spatial autocorrelation calculations of supply and demand, and K-means cluster analysis of five demand indicators at each hospital site, to further reveal the quantitative and spatial differences between each cluster. Although the coupling coordination results showed that supply was already higher than demand, the results were only a numerical match in the mathematical model, and there was still a gap between the number of medical resources in Shenzhen and equivalent large cities. For example, according to existing studies, Beijing and Shanghai led the inter-provincial coordination of resident health and investment in health resources [53]. In Beijing, the number of licensed physicians per 1000 people and the number of actual beds per 1000 people were 5.08 and 5.73, respectively, which exceeded comparable indicators for Shenzhen in the same period (2.79 and 3.65, respectively). Despite the outstanding achievements of the medical reforms, they were still unable to achieve a balanced spatial allocation of medical resources. The system also failed to meet the personalized medical needs of residents, and there is a need to enhance the quality of medical resources while ensuring that a comprehensive reform of the medical and health system is undertaken.

### 4.3. Policy Recommendations for Resource Management

Overall, against the backdrop of economic development and rising social security levels, Shenzhen’s medical resources were under pressure to provide services in the face of increasing demand. Based on our conclusions, the following suggestions are provided: the administrative regions of Shenzhen should take advantage of the historical opportunity of building a pilot demonstration area of socialism with Chinese characteristics (Table A4) in Shenzhen, so as to reasonably allocate medical resources while considering the specific economic and population situation observed in each region. In terms of human resources, there is a need to vigorously introduce high-end talent resources, and to formulate policies that ensure a match between medical talent and Shenzhen’s people-in policy, while also encouraging scarce talent to remain in Shenzhen. With respect to medical institutions, in addition to introducing high-level disciplinary teams, medical schools, and brand-name hospitals, it is important to ensure the rational planning of medical institution sites in order to narrow the gap that exists between regions [54]. In terms of technological application, there is a need to promote the development of a big-data health application, encourage the widespread sharing of medical resource information between regions, and support the construction of a smart medical care system, while also achieving online and offline integration of the hospital system, so as to improve the efficiency of the use of medical resources.

### 4.4. Limitations and Improvements for Future Research

There were three limitations in this study. First, instead of conducting the accessibility evaluation by hospital tier, as in other medical resource accessibility studies, this study measured the overall medical resource carrying capacity of public hospitals in Shenzhen and included hospital tiers as a indicator in the evaluation system. Second, regarding the research object, the medical resources included in the research scope on the supply side were public hospitals. This was because there are no detailed medical resource indicators for community healthcare centers (Table A4) and private hospitals, and the capacity and scope of their services are not very clear [50,51,55]. Therefore, they were not included in the carrying capacity calculation. The definition of medical resources only considered public hospitals. Third, considering the construction of the model, the parameter-setting of the coupling coordination degree should strive for greater generalizability when measuring the supply of and demand for medical resources. Finally, the demand indicators were measured by reference to population size, which is rather general.

Future work can be carried out in four ways:

1. Expand the inclusion criteria to public hospitals, socially-run hospitals, and social recreation. The demand indicators can be further divided according to the diverse needs of residents. For example, the population could be classified by gender and age, or an in-depth investigation could be carried out in line with the changing disease spectrum, or by focusing on rehabilitation, nursing, and convalescence.
2. After completing the research object, according to the carrying capacity of specific hospital points and people’s demand for medical resources, the supply and demand matching of medical resources in various regions of Shenzhen could be obtained, which would make the research results more specific and provide more scientific information for the government to reasonably plan medical resources.
3. The grey relational analysis method [56] could be used to calculate the grey relational degree between each index and the carrying capacity within the two dimensions of supply and demand, so as to reveal the level of correlation between each system and reveal the key indexes affecting the carrying capacity.
4. In future studies, with more data, the carrying capacity evaluation of hospitals within the hierarchy could be conducted.

## 5. Conclusions

This study focused on the supply of and demand for medical resources in Shenzhen, and constructed an index system to assess their coordination. Applying mobile signaling data, the carrying capacity was quantified based on the 2SFCA method. This paper examined the spatial distribution of supply and demand, and performed time-series variation of the coupling coordination degree of this supply-and-demand system by using spatial autocorrelation analysis and the coupling coordination degree model. The main conclusions obtained were as follows.

1. The carrying capacity and supply level of medical resources in Shenzhen showed large spatial differences. Areas that had a higher carrying capacity for medical resources were distributed in the central and southeastern regions, while a lower carrying capacity was found along the northwestern, northern, and southern edges. Hospitals with higher supply levels were distributed in the southwestern part of Shenzhen, namely, the southern part of Nanshan District, Futian District, and Luohu District. Areas with lower supply levels were situated in the western part of eastern Shenzhen. Overall, a pattern of uneven distribution was observed both inside and outside the former Special Administrative Region.
2. From 1986 to 2019, the overall level of the supply of and demand for medical resources in Shenzhen increased, and the coupling coordination degree between supply and demand also continued to rise. In 2019, the coordination status of supply and demand was still on the verge of disorder until 2019, although it showed an overall trend towards coordinated development. Since 2005, the level of supply has remained slightly higher than the level of demand, which indicates that supply has continued to exceed demand.
3. The spatial match between the supply of and demand for medical resources was more consistent in the central-western and eastern parts of Shenzhen, such that the clearest high-demand area was located in the central-western part of Shenzhen, which was in line with its high population density and well-developed road network.
4. The four eastern administrative districts of Shenzhen (Longgang District, Yantian District, Pingshan District and Dapeng New District) were not as rich in medical resources as others, so new public hospitals should be laid out in these districts as a priority. In the low-high agglomeration area, the supply capacity of medical resources was not sufficient, so we should focus on upgrading hospitals or setting up new hospitals in this area, while maintaining the existing medical resources in the high-high agglomeration area. We might consider upgrading hospitals or building new hospitals as appropriate, and maintaining the existing level of medical resource supply in the high-low and low-low agglomeration areas.

## Figures and Tables

**Table 1:** Assessment index system: Coordination of the supply of and demand for medical resources in Shenzhen.

Element Layer	Factor Level	Weight	Index Attribute
Supply level	Health care institutions (pcs)	0.0842	Positive
	Total hospitals (pcs)	0.0454	Positive
	The number of beds (pcs)	0.0725	Positive
	Licensed doctors (pcs)	0.0712	Positive
	Registered nurses (person)	0.0882	Positive
	Pharmacists (person)	0.0570	Positive
	Laboratory technicians (person)	0.0705	Positive
	Gross domestic product (10,000 yuan)	0.1095	Positive
Demand level	Total patients treated (10,000 persons)	0.0805	Positive
	Total inpatients (10,000 persons)	0.0837	Positive
	Utilization rate of beds (%)	0.0265	Positive
	Turnover of beds (time)	0.0370	Positive
	Year-end permanent population (10,000 persons)	0.0402	Positive
	Hospital beds per 1000 persons (pcs)	0.0444	Positive
	Physicians per 1000 persons (person)	0.0360	Positive
	Hospital beds per 1000 persons (pcs)	0.0533	Positive
	Per capita disposable income (10,000 yuan)	0.0842	Positive

**Table 2:** Coupling coordination degree model formula.

Geographic Model	Calculation Formula	Model Interpretation	Meaning
Coordination degree of supply and demand level	$C={\left[\left(\frac{X_{i}\times Y_{i}}{{\left(\left(X_{i}+Y_{i}\right)∕2\right)}^{2}}\right)\right]}^{k}$	*C* represents the coupling degree. *k* is the adjustment coefficient, the study takes *k* = 2 [44].	The larger *C*, the better the coupling between supply and demand.
Comprehensive evaluation index	$T=\alpha \times x_{i}+\beta \times Y_{i}$	*T* represents the comprehensive evaluation index to reflect the level of supply and demand level of medical resources; *α* and *β* are undetermined coefficients.	*T* reflects the overall level of medical resources. When supply is as important as the demand coefficient, the study takes *α* = *β* = 0.5.
Coupling coordination degree method	$D=\sqrt{C\times T}$	*D* is the CCD between supply and EE, and the value of *D* is between 0 and 1.	The larger the value of *D*, the more harmonious the coupling relationship between the supply and demand.

**Table 3:** The grade classification of the coordinated development degree.

Coupling Coordination Degree	0–0.30	0.3–0.4	0.4–0.5	0.5–0.6	0.6–0.7	0.7–0.8	0.8–0.9	0.9–1.0
Coordination level	Disordered	On the verge of disorder	Reluctantly coordinated	Low coordination	Primary coordination	Middle coordination	Good coordination	Better coordination

**Table 4:** Carrying capacity index classification statistics.

Carrying Capacity Level	Carrying Capacity Index	The Number of TAZ	Percentage
Lower	0.055–0.155	231	23.89%
Low	0.155–0.255	269	27.82%
Middle	0.255–0.355	156	16.13%
High	0.35–0.455	231	23.89%
Higher	0.455–0.855	80	8.27%
Total	/	967	100.00%

## Data Availability

Not applicable.

## Abbreviations

SEZ	Shenzhen Special Economic Zone
TAZ	traffic analysis zone
OD	origin–destination

## Appendix A

**Table A1:** The change in the coupling coordination degree of the supply and demand from 1986 to 2019.

Year	Supply Level	Demand Level	Coupling Coordination Degree
1986	0.00006	0.04449	0.03397
1987	0.00363	0.03852	0.02284
1988	0.00794	0.04217	0.04221
1989	0.01125	0.04074	0.05469
1990	0.01581	0.04916	0.06637
1991	0.02017	0.05471	0.07617
1992	0.02749	0.05901	0.09017
1993	0.03461	0.05714	0.10064
1994	0.04423	0.04831	0.10734
1995	0.05706	0.04843	0.11407
1996	0.08440	0.06226	0.13231
1997	0.08579	0.07100	0.13875
1998	0.08476	0.07944	0.14311
1999	0.08295	0.07791	0.14166
2000	0.08837	0.10519	0.15438
2001	0.09669	0.11519	0.16150
2002	0.10624	0.12597	0.16914
2003	0.12517	0.14829	0.18357
2004	0.13704	0.16085	0.19173
2005	0.15965	0.14344	0.19409
2006	0.23799	0.17372	0.22133
2007	0.24880	0.19829	0.23338
2008	0.26653	0.21562	0.24276
2009	0.28500	0.22655	0.24957
2010	0.29130	0.24446	0.25681
2011	0.31377	0.26554	0.26723
2012	0.34009	0.27403	0.27386
2013	0.37125	0.28319	0.28084
2014	0.39785	0.29095	0.28636
2015	0.43001	0.30514	0.29440
2016	0.47401	0.32848	0.30630
2017	0.50704	0.34517	0.31461
2018	0.54918	0.36179	0.32317
2019	0.59856	0.39110	0.33627

**Table A2:** The supply of Shenzhen’s medical resource carrying capacity.

Year	Health Care Institutions /pcs	Total Hospitals /pcs	Number of Beds /pcs	Licensed Doctors /Person	Registered Nurses /Person	Pharmacists /Person	Laboratory Technicians /Person	Gross Domestic Product /10,000 Yuan
1986	297	32	2112	2217	1246	260	211	416,451
1987	311	34	2309	2408	1450	292	257	559,015
1988	308	35	2580	2754	1734	409	283	869,807
1989	332	35	2922	3103	1971	431	321	1,156,565
1990	354	38	3192	3426	2145	475	355	1,716,665
1991	360	41	3582	3737	2372	539	365	2,366,630
1992	397	45	4550	4247	2705	630	375	3,173,194
1993	400	45	5252	4798	3225	744	464	4,531,445
1994	496	48	6124	5347	3625	823	538	6,346,711
1995	506	63	6724	6050	4034	906	612	8,427,933
1996	1422	65	7455	7266	4654	1005	664	10,505,121
1997	1126	72	8288	7400	4828	1063	708	13,023,008
1998	899	72	8899	7191	5025	1111	760	15,449,472
1999	687	71	9332	7062	5230	1108	812	18,246,876
2000	683	72	10,294	7418	5425	1130	847	22,192,015
2001	723	75	11,159	8097	5945	1194	871	25,229,474
2002	761	77	12,404	7853	6635	1292	965	30,172,384
2003	893	85	13,588	8909	7321	1499	1085	36,401,435
2004	856	87	15,069	9846	7975	1569	1219	43,502,928
2005	1063	97	16,824	10,961	8981	1710	1362	50,357,678
2006	1692	99	17,553	16,238	16,583	2796	2340	59,206,612
2007	1781	101	18,086	17,450	17,869	2876	2190	69,252,268
2008	1806	100	19,913	18,807	19,339	2979	2382	79,414,328
2009	1963	101	21,399	19,963	21,008	3046	2578	85,144,707
2010	1827	107	22,842	20,122	21,866	2896	2486	100,690,553
2011	1854	110	24,079	21,517	23,987	3021	2615	119,228,085
2012	2008	115	27,984	22,831	25,931	3143	2652	134,962,675
2013	2228	117	29,261	24,221	28,035	3343	3022	152,342,436
2014	2532	122	31,042	25,728	29,723	3469	3086	167,953,507
2015	2946	123	33,771	27,834	31,717	3607	3216	184,368,359
2016	3339	134	41,512	29,300	34,065	3644	3364	206,857,358
2017	3492	135	43,868	31,838	36,389	3842	3517	232,802,719
2018	3806	139	47,551	34,747	40,309	4029	3739	252,660,771
2019	4459	144	51,318	38,519	44,273	4210	3934	269,270,920

**Table A3:** The demand of Shenzhen’s medical resource carrying capacity.

Year	Total Patients Treated /10,000 Persons	Total Inpatients /10,000 Persons	Utilization Rate of Beds (%)	Turnover of Beds (Time)	Year-end Permanent Population /10,000 Persons	Hospital Beds per 1000 Persons/pcs	Physicians per 1000 Persons/pcs	Per Capita Disposable Income of Residents /10,000 Yuan
1986	389	4.68	66.2	24.4	93.56	2.26	2.37	0.1817
1987	461	5.84	73.6	22.2	105.44	2.00	2.09	0.2091
1988	563	6.97	75.6	28.8	120.14	1.68	1.80	0.2569
1989	607	7.87	78.4	29	141.6	1.53	1.62	0.3657
1990	729	8.73	81.4	29	167.78	1.58	1.70	0.4127
1991	840	10.08	85.2	30.3	226.76	1.50	1.57	0.4564
1992	976	11.4	81.9	28	268.02	1.74	1.63	0.7737
1993	1050	12.59	80.3	26.6	335.97	1.78	1.63	0.5783
1994	1057	12.81	70.8	23.2	412.71	1.83	1.59	1.0503
1995	1202	13.07	65	20.8	449.15	1.95	1.75	1.2771
1996	1168	14.63	64.3	21.5	482.89	2.08	2.03	1.6296
1997	1231	16.15	67.4	21.9	527.75	2.18	1.95	1.8579
1998	1386	19.05	68.6	24.1	580.33	2.25	1.82	1.9214
1999	1520	21.12	69.3	24.5	632.56	2.30	1.74	1.252
2000	1699	26.61	75.4	28.3	701.24	2.38	1.71	2.0906
2001	1912	30.23	77.3	29.4	724.57	2.38	1.73	2.276
2002	2062	35.74	80	30.6	746.62	2.46	1.64	2.4941
2003	3052	41.57	84	33.5	778.27	2.44	1.69	2.5936
2004	3514	49	82.4	34.9	800.8	2.52	1.73	2.7596
2005	4055	54.68	79.9	34.6	827.75	2.03	1.40	2.1494
2006	5170	59.24	81.6	35.6	871.1	2.02	2.02	2.2567
2007	5954	68.09	87.5	38.6	912.37	1.98	2.06	2.4301
2008	6842	75.34	89.1	37.9	954.28	2.09	2.11	2.6729
2009	7549	79.7	86.1	38.4	995.01	2.15	2.15	2.9245
2010	8127	89.1	89.2	40.1	1037.2	2.2	2.05	3.2381
2011	8878	96.2	90.5	41.4	1046.74	2.3	2.16	3.6505
2012	8638	105.4	85.8	40.1	1054.74	2.65	2.27	4.0742
2013	9112	109.4	84.2	38.4	1062.89	2.75	2.39	4.4653
2014	8853	119.4	85.5	39.4	1077.89	2.88	2.49	4.0948
2015	8901	124.1	84	38.1	1137.87	3.35	2.55	4.4633
2016	9598	138.4	85.2	39	1190.84	3.49	2.57	4.8695
2017	9960	150.8	84.8	39.3	1252.83	3.5	2.66	5.2938
2018	9986	162	84	39.6	1302.66	3.65	2.79	5.7544
2019	10,812	182.2	82.5	40.1	1343.88	3.83	3.01	6.2522

**Table A4:** Explanation of specific words in the text.

Specific Vocabulary	Explanation
Community Health Centers	To integrate urban healthcare resources, China began to construct a two-level healthcare system consisting of tertiary hospitals (large general hospitals) and community health centers in 1997 [57]. Community health centers refer to the health care activities of prevention, medical treatment, rehabilitation, and health promotion provided by health and related departments to residents in a certain community. They also play an important role in primary health screening and management globally in defending against the 2019 novel coronavirus (COVID-19) [58].
Pilot Demonstration Area of Socialism with Chinese Characteristics	The Central Committee of the Communist Party of China and the State Council issued relevant documents in August 2019, elevating Shenzhen to the strategic status of being a model city for the rule of law in China, among the top global cities, and a city example for building a strong, modern, socialist state in China [59].
